# Draft Genome Sequence of *Frankia* Strain G2, a Nitrogen-Fixing Actinobacterium Isolated from *Casuarina equisetifolia* and Able To Nodulate Actinorhizal Plants of the Order *Rhamnales*

**DOI:** 10.1128/genomeA.00437-16

**Published:** 2016-05-26

**Authors:** Imen Nouioui, Maher Gtari, Markus Göker, Faten Ghodhbane-Gtari, Louis S. Tisa, Maria P. Fernandez, Philippe Normand, Marcel Huntemann, Alicia Clum, Manoj Pillay, Neha Varghese, T. B. K. Reddy, Natalia Ivanova, Tanja Woyke, Nikos C. Kyrpides, Hans-Peter Klenk

**Affiliations:** a School of Biology, Newcastle University, Newcastle upon Tyne, United Kingdom; b University of Tunis-El Manar, Tunis, Tunisia; c Leibniz Institute-DSMZ, Braunschweig, Germany; d University of New Hampshire, Durham, New Hampshire, USA; e Université de Lyon, CNRS, Ecologie Microbienne, INRA, UMR1418, Villeurbanne, France; f DOE Joint Genome Institute, Walnut Creek, California, USA

## Abstract

*Frankia* sp. strain G2 was originally isolated from *Casuarina equisetifolia* and is characterized by its ability to nodulate actinorhizal plants of the *Rhamnales* order, but not its original host. It represents one of the largest *Frankia* genomes so far sequenced (9.5 Mbp).

## GENOME ANNOUNCEMENT

The genus *Frankia* contains actinobacteria known for their ability to fix nitrogen and to infect the roots of eight actinorhizal plant families (1–3). Phylogenetic studies of *Frankia* strains based on 16S rRNA (4), *gyr* B (5), *gln II* (5, 6) genes and 16S-23S rRNA Intergenic Spacer Region (7) indicate four groups. Group 1 forms nodules on *Betulaceae*, *Myricaceae*, and *Casuarinaceae*. Group 2 contains microsymbionts of *Coriariaceae*, *Datiscaceae*, *Dryadoideae* (*Rosaceae*), and *Ceanothus* (*Rhamnaceae*). Group 3 includes strains associated with *Morella* (*Myricaceae*), *Colletieae* (*Rhamnaceae*), *Elaeagnaceae*, and *Gymnostoma* (*Casuarinaceae*). Group 4 includes atypical, non-infective (Nod-) and/or ineffective (Fix-) *Frankia* strains. Our knowledge about the biology of this genus has been well improved due to the information provided by sequenced *Frankia* genomes (8–21). Group 3 has a broad host range, considerable genetic diversity between the strains (5, 7), high potential for a saprophytic lifestyle (7–22), and a variable genome size ranging from 7.5 to 10.45 Mbp. Strain G2 (=DSM45899=CECT9038) was selected for genome sequencing within the Genomic Encyclopaedia of Type Strains, Phase II: From Individual Species to Whole Genera (23), the second production phase of the Genomic Encyclopedia of *Bacteria* and *Archaea*: Sequencing a Myriad of Type Strains Initiative (24). The candidate type strain G2 for a novel *Frankia* species, selected to enrich the diversity of group 3, was isolated from *Casuarina equisetifolia* nodules collected in the INRA Research Station, Saint-François, Grande Terre, Guadeloupe (25). It has the potential to produce natural products such as the red-pigmented antibiotics (benzo[a]naphthacenequinones) (26). It is infective on members of the actinorhizal *Rhamnales*, but not on its original host plant *C. equisetifolia* (25). The draft genome of strain G2 was sequenced using Illumina technology (27) with a 300 bp insert standard shotgun library on an Illumina HiSeq-2500 1-TB platform, which generated 6,201,478 reads totaling 936.4 Mbp, at the Joint Genome Institute (JGI) (28). The assembly was realized using Velvet (version 1.2.07) (29) and Allpaths-LG (version r46652) (30). Annotation was performed using the JGI annotation pipeline (31) and the data are available from the IMG data management system (32). The final draft assembly contained 90 contigs in 83 scaffolds, totaling 9.537,992 bp in size based on 856.6 Mbp of data with 171.3 × input read coverage. The genome draft encodes 7,790 protein genes, 47 tRNAs, and 2 rRNA regions, with an overall G+C content of 70.9%. Genome annotation was performed as described by Tisa et al. (21). As expected, since *Frankia* is a nitrogen fixing actinobacterium, six nitrogenase genes, *nifH*, *nifE, nifD, nifK, nifW*, and *nifN*, have been detected.

Project information is available in the Genomes Online Database (33) and DNA from the DNA Bank Network (34).

### Nucleotide sequence accession number.

This whole-genome shotgun project has been deposited in DDBJ/EMBL/GenBank under the accession no. FAOZ00000000. The version described in this paper is the first version.

## References

[1] Benson DR, Silvester WB 1993 Biology of *Frankia* strains, actinomycete symbionts of actinorhizal plants. Microbiol Rev 57:293–319. 8336669 10.1128/mr.57.2.293-319.1993 PMC372911

[2] Schwencke J, Carú M 2001 Advances in actinorhizal symbiosis: host plant-*Frankia* interactions, biology, and applications in arid land reclamation. Arid Land Res Manag 15:285–327. doi:10.1080/153249801753127615.

[3] Chaia EE, Wall LG, Huss-Danell K 2010 Life in soil by actinorhizal root nodule endophyte *Frankia*. A review. Symbiosis 51:201–226. doi:10.1007/s13199-010-0086-y.

[4] Normand P, Orso S, Cournoyer B, Jeannin P, Chapelon C, Dawson J, Evtushenko L, Misra AK 1996 Molecular phylogeny of the genus *Frankia* and related genera and emendation of the family *Frankiaceae*. Int J Syst Bacteriol 46:1–9. doi:10.1099/00207713-46-1-1. 8573482

[5] Nouioui I, Ghodhbane-Gtari F, Beauchemin NJ, Tisa LS, Gtari M 2011 Phylogeny of members of the *Frankia* genus based on *gyrB*, *nifH* and *glnII* sequences. Antonie Van Leeuwenhoek 100:579–587. doi:10.1007/s10482-011-9613-y. 21713368

[6] Cournoyer B, Lavire C 1999 Analysis of *Frankia* evolution radiation using *glnII* sequences. FEMS Microbiol Lett 177:29–34. 10436920 10.1111/j.1574-6968.1999.tb13709.x

[7] Ghodhbane-Gtari F, Nouioui I, Chair M, Boudabous A, Gtari M 2010 16S-23S rRNA intergenic spacer region variability in the genus *Frankia*. Microb Ecol 60:487–495. doi:10.1007/s00248-010-9641-6 20179918

[8] Gtari M, Ghodhbane-Gtari F, Nouioui I, Ktari A, Hezbri K, Mimouni W, Sbissi I, Ayari A, Yamanaka T, Normand P, Tisa LS, Boudabous A 2015 Cultivating the uncultured: growing the recalcitrant cluster-2 *Frankia* strains. Sci Rep 5:13112. doi:10.1038/srep13112. 26287281 PMC4541404

[9] Normand P, Lapierre P, Tisa LS, Gogarten JP, Alloisio N, Bagnarol E, Bassi CA, Berry AM, Bickhart DM, Choisne N, Couloux A, Cournoyer B, Cruveiller S, Daubin V, Demange N, Francino MP, Goltsman E, Huang Y, Kopp OR, Labarre L, Lapidus A, Lavire C, Marechal J, Martinez M, Mastronunzio JE, Mullin BC, Niemann J, Pujic P, Rawnsley T, Rouy Z, Schenowitz C, Sellstedt A, Tavares F, Tomkins JP, Vallenet D, Valverde C, Wall LG, Wang Y, Medigue C, Benson DR 2007 Genome characteristics of facultatively symbiotic *Frankia* sp. strains reflect host range and host plant biogeography. Genome Res 17:7–15. 17151343 10.1101/gr.5798407 PMC1716269

[10] Normand P, Queiroux C, Tisa LS, Benson DR, Rouy Z, Cruveiller S, Médigue C 2007 Exploring the genomes of *Frankia*. Physiol Plant 130:331–343. doi:10.1111/j.1399-3054.2007.00918.x.

[11] Persson T, Benson DR, Normand P, VandenHeuvel B, Pujic P, Chert-kov O, Teshima H, Bruce DC, Detter C, Tapia R, Han S, Han J, Woyke T, Pitluck S, Pennacchio L, Nolan M, Ivanova N, Pati A, Land ML, Pawlowski K, Berry AM 2011 Genome sequence of “*Candidatus Frankia datiscae*” Dg1, the uncultured microsymbiont from nitrogen-fixing root nodules of the dicot *Datisca glomerata*. J Bacteriol 193:7017–7018. doi:10.1128/JB.06208-11. 22123767 PMC3232863

[12] Ghodhbane-Gtari F, Beauchemin N, Bruce D, Chain P, Chen A, Davenport KW, Deshpande S, Detter C, Furnholm T, Goodwin L, Gtari M, Han C, Han J, Huntemann M, Ivanova N, Kyrpides NC, Land ML, Markowitz V, Mavrommatis K, Nolan M, Nouioui I, Pagani I, Pati A, Pitluck S, Santos CL, Sen A, Sur S, Szeto E, Tavares F, Teshima H, Thakur S, Wall LG, Woyke T, Tisa LS 2013 Draft genome sequence of *Frankia* sp. strain CN3, an atypical, non-infective (Nod) ineffective (Fix) isolate from *Coriaria nepalensis*. Genome Announc 1(2):e00085-13. 23516212 10.1128/genomeA.00085-13 PMC3622958

[13] Sen A, Beauchemin N, Bruce D, Chain P, Chen A, Walston Davenport K, Deshpande S, Detter C, Furnholm T, Ghodbhane-Gtari F, Goodwin L, Gtari M, Han C, Han J, Huntemann M, Ivanova N, Kyrpides N, Land ML, Markowitz V, Mavrommatis K, Nolan M, Nouioui I, Pagani I, Pati A, Pitluck S, Santos CL, Sur S, Szeto E, Tavares F, Teshima H, Thakur S, Wall LG, Woyke T, Tisa LS 2013 Draft genome sequence of *Frankia* sp. strain QA3, a nitrogen-fixing actinobacterium isolated from the root nodule of *Alnus nitida*. Genome Announc 1(2):e00103-13. doi:10.1128/genomeA.00103-13. 23516220 PMC3622976

[14] Nouioui I, Beauchemin N, Cantor MN, Chen A, Detter JC, Furnholm T, Ghodhbane-Gtari F, Goodwin L, Gtari M, Han C, Han J, Huntemann M, Hua SX, Ivanova N, Kyrpides N, Markowitz V, Mavrommatis K, Mikhailova N, Nordberg HP, Ovchinnikova G, Pagani I, Pati A, Sen A, Sur S, Szeto E, Thakur S, Wall LG, Wei C-L, Woyke T, Tisa LS 2013 Draft genome sequence of *Frankia* sp. strain BMG5.12, a nitrogen-fixing actinobacterium isolated from Tunisian soils. Genome Announc 1(4):e00468-13. doi:10.1128/genomeA.00468-13. 23846272 PMC3709149

[15] Wall LG, Beauchemin N, Cantor MN, Chaia E, Chen A, Detter JC, Furnholm T, Ghodhbane-Gtari F, Goodwin L, Gtari M, Han C, Han J, Huntemann M, Hua SX, Ivanova N, Kyrpides N, Markowitz V, Mavrommatis K, Mikhailova N, Nordberg HP, Nouioui I, Ovchinnikova G, Pagani I, Pati A, Sen A, Sur S, Szeto E, Thakur S, Wei C-L, Woyke T, Tisa LS 2013 Draft genome sequence of *Frankia* sp. strain BCU110501, a nitrogen-fixing actinobacterium isolated from nodules of *Discaria trinevis*. Genome Announc 1(4):e00503-13. doi:10.1128/genomeA.00503-13. 23846281 PMC3709158

[16] Mansour SR, Oshone R, Hurst SG IV, Morris K, Thomas WK, Tisa LS 2014 Draft genome sequence of *Frankia* sp. strain CcI6, a salt-tolerant nitrogen-fixing actinobacterium isolated from the root nodule of *Casuarina cunninghamiana*. Genome Announc 2(1):e01205-13. doi:10.1128/genomeA.01205-13. 24435877 PMC3894291

[17] Hurst SG IV, Oshone R, Ghodhbane-Gtari F, Morris K, Abebe-Akele F, Thomas WK, Ktari A, Salem K, Mansour S, Gtari M, Tisa LS 2014 Draft genome sequence of *Frankia* sp. strain Thr, a nitrogen-fixing actinobacterium isolated from the root nodules of *Casuarina cunninghamiana* grown in Egypt. Genome Announc 2(3):e00493-14. doi:10.1128/genomeA.00493-14. 24855310 PMC4031348

[18] Ghodhbane-Gtari F, Hurst SG IV, Oshone R, Morris K, Abebe-Akele F, Thomas WK, Ktari A, Salem K, Gtari M, Tisa LS 2014 Draft genome sequence of *Frankia* sp. strain BMG5.23, a salt-tolerant nitrogen-fixing actinobacterium isolated from the root nodules of *Casuarina glauca* grown in Tunisia. Genome Announc 2(3):e00520-14. doi:10.1128/genomeA.00520-14. 24874687 PMC4038892

[19] Tisa LS, Beauchemin N, Gtari M, Sen A, Wall LG 2013 What stories can the *Frankia* genomes start to tell us? J Biosci 38:719–726. doi:10.1007/s12038-013-9364-1. 24287651

[20] Pujic P, Bolotin A, Fournier P, Sorokin A, Lapidus A, Richau KH, Briolay J, Mebarki F, Normand P, Sellstedt A 2015 Genome sequence of the atypical symbiotic *Frankia* R43 strain, a nitrogen-fixing and hydrogen-producing actinobacterium. Genome Announc 3(6):e01387-15. doi:10.1128/genomeA.01387-15. 26607894 PMC4661313

[21] Tisa LS, Beauchemin N, Cantor MN, Furnholm T, Ghodhbane-Gtari F, Goodwin L, Copeland A, Gtari M, Huntemann M, Ivanova N, Kyrpides N, Markowitz V, Mavrommatis K, Mikhailova N, Nouioui I, Oshone R, Ovchinnikova G, Pagani I, Palaniappan K, Pati A, Sen A, Shapiro N, Szeto E, Wall L, Wishart J, Woyke T 2015 Draft genome sequence of *Frankia* sp. strain DC12, an atypical, noninfective, ineffective isolate from *Datisca cannabina*. Genome Announc 3(4):e00889-15. doi:10.1128/genomeA.00889-15. 26251504 PMC4541282

[22] Benson DR, Vanden Heuvel BD, Potter D 2004 Actinorhizal symbioses: diversity and biogeography: Plant06, p. 99–129. In Gillings M, Holmes A (ed.), Plant microbiology. BIOS Scientific Publishers, Oxford.

[23] Wu D, Hugenholtz P, Mavromatis K, Pukall R, Dalin E, Ivanova NN, Kunin V, Goodwin L, Wu M, Tindall BJ, Hooper SD, Pati A, Lykidis A, Spring S, Anderson IJ, D’haeseleer P, Zemla A, Singer M, Lapidus A, Nolan M, Copeland A, Han C, Chen F, Cheng JF, Lucas S, Kerfeld C, Lang E, Gronow S, Chain P, Bruce D, Rubin EM, Kyrpides NC, Klenk HP, Eisen JA 2009 A phylogeny-driven genomic encyclopaedia of *Bacteria* and *Archaea*. Nature 462:1056–1060. doi:10.1038/nature08656. 20033048 PMC3073058

[24] Kyrpides NC, Hugenholtz P, Eisen JA, Woyke T, Göker M, Parker CT, Amann R, Beck BJ, Chain PSG, Chun J, Colwell RR, Danchin A, Dawyndt P, Dedeurwaerdere T, DeLong EF, Detter JC, De Vos P, Donohue TJ, Dong XZ, Ehrlich DS, Fraser C, Gibbs R, Gilbert J, Glina P, Glöckner FO, Jansson JK, Keasling JD, Knight R, Labeda D, Lapidus A, Lee JS, Li WJ, Juncai MA, Markowitz V, Edward RBM, Morrison M, Meyer F, Nelson KE, Ohkuma M, Ouzounis CA, Pace N, Parkhill J, Qin N, Rossello-Mora R, Sikorski J, Smith D, Sogin M, Stevens R, Stingl U, Suzuki KI, Taylor D, Tiedje JM, Tindall B, Wagner M, Weinstock G, Weissenbach J, White O, Wang J, Zhang L, Zhou YG, Field D, Whitman WB, Garrity GM, Klenk HP 2014 Genomic encyclopedia of *Bacteria* and *Archaea*: sequencing a myriad of type strains. PLoS Biol 12:e1001920. doi:10.1371/journal.pbio.1001920. 25093819 PMC4122341

[25] Diem HG, Gauthier D, Dommergues YR 1982 Isolation of *Frankia* from nodules of *Casuarina equisetifolia*. Can J Microbiol 28:526–530. doi:10.1139/m82-079.

[26] Gerber NN, Lechevalier MP 1984 Novel benzo[a]naphthacenequinones from an actinomycete, *Frankia* 6–2 (ORS 020604). Can J Chem 62:2818–2821.

[27] Bennett S 2004 Solexa Ltd. Pharmacogenomics 5:433–438. doi:10.1517/14622416.5.4.433. 15165179

[28] Mavromatis K, Land ML, Brettin TS, Quest DJ, Copeland A, Clum A, Goodwin L, Woyke T, Lapidus A, Klenk HP, Cottingham RW, Kyrpides NC 2012 The fast changing landscape of sequencing technologies and their impact on microbial genome assemblies and annotation. PLoS One 7:e48837. doi:10.1371/journal.pone.0048837. 23251337 PMC3520994

[29] Zerbino DR, Birney E 2008 Velvet: algorithms for *de novo* short read assembly using de Bruijn graphs. Genome Res 18:821–829. doi:10.1101/gr.074492.107. 18349386 PMC2336801

[30] Gnerre S, MacCallum I, Przybylski D, Ribeiro FJ, Burton JN, Walker BJ, Sharpe T, Hall G, Shea TP, Sykes S, Berlin AM, Aird D, Costello M, Daza R, Williams L, Nicol R, Gnirke A, Nusbaum C, Lander ES, Jaffe DB 2011 High-quality draft assemblies of mammalian genomes from massively parallel sequence data. Proc Natl Acad Sci USA 108:1513–1518. doi:10.1073/pnas.1017351108. 21187386 PMC3029755

[31] Huntemann M, Ivanova NN, Mavromatis K, Tripp HJ, Paez-Espino D, Palaniappan K, Szeto E, Pillay M, Chen IM-A, Pati A, Nielsen T, Markowitz VM, Kyrpides NC 2015 The Standard operating procedure of the DOE-JGI microbial genome annotation pipeline (MGAP v.4). Stand Genomic Sci 10:86. 26512311 10.1186/s40793-015-0077-y PMC4623924

[32] Markowitz VM, Chen IM, Palaniappan K, Chu K, Szeto E, Pillay M, Ratner A, Huang J, Woyke T, Huntemann M, Anderson I, Billis K, Varghese N, Mavromatis K, Pati A, Ivanova NN, Kyrpides NC 2014 IMG 4 version of the integrated microbial genomes comparative analysis system. Nucleic Acids Res 42:D560–D567. doi:10.1093/nar/gkt963. 24165883 PMC3965111

[33] Reddy TB, Thomas AD, Stamatis D, Bertsch J, Isbandi M, Jansson J, Mallajosyula J, Pagani I, Lobos EA, Kyrpides NC 2015 The Genomes Online Database (GOLD) v.5: a metadata management system based on a four level (meta) genome project classification. Nucleic Acids Res 43:D1099–D1106. doi:10.1093/nar/gku950. 25348402 PMC4384021

[34] Gemeinholzer B, Dröge G, Zetzsche H, Haszprunar G, Klenk HP, Güntsch A, Berendsohn WG, Wägele JW 2011 The DNA bank network: the start from a German initiative. Biopreserv Biobank 9:51–55. doi:10.1089/bio.2010.0029. 24850206

